# Summed Probability Distribution of ^14^C Dates Suggests Regional Divergences in the Population Dynamics of the Jomon Period in Eastern Japan

**DOI:** 10.1371/journal.pone.0154809

**Published:** 2016-04-29

**Authors:** Enrico R. Crema, Junko Habu, Kenichi Kobayashi, Marco Madella

**Affiliations:** 1 McDonald Institute for Archaeological Research, University of Cambridge, Cambridge, United Kingdom; 2 CaSEs Research Group (Complexity and Socio-Ecological Dynamics), Department of Humanities, Pompeu Fabra University, Barcelona, Spain; 3 UCL Institute of Archaeology, London, United Kingdom; 4 Research Institute of for Humanity and Nature, Kyoto, Japan; 5 Dept. of Anthropology, University of California, Berkeley, California, United States of America; 6 Faculty of Letters, Chuo University, Tokyo, Japan; 7 IMF-CSIC, Barcelona, Spain; 8 ICREA, Barcelona, Spain; University College Dublin, IRELAND

## Abstract

Recent advances in the use of summed probability distribution (SPD) of calibrated ^14^C dates have opened new possibilities for studying prehistoric demography. The degree of correlation between climate change and population dynamics can now be accurately quantified, and divergences in the demographic history of distinct geographic areas can be statistically assessed. Here we contribute to this research agenda by reconstructing the prehistoric population change of Jomon hunter-gatherers between 7,000 and 3,000 cal BP. We collected 1,433 ^14^C dates from three different regions in Eastern Japan (Kanto, Aomori and Hokkaido) and established that the observed fluctuations in the SPDs were statistically significant. We also introduced a new non-parametric permutation test for comparing multiple sets of SPDs that highlights point of divergences in the population history of different geographic regions. Our analyses indicate a general rise-and-fall pattern shared by the three regions but also some key regional differences during the 6^th^ millennium cal BP. The results confirm some of the patterns suggested by previous archaeological studies based on house and site counts but offer statistical significance and an absolute chronological framework that will enable future studies aiming to establish potential correlation with climatic changes.

## Introduction

The Jomon culture of Japan (ca 16,000 ~ 2,500 cal BP) is often regarded as a prime example of a parallel evolutionary pathway where traits commonly associated with farming societies have been independently developed within a primarily hunter-gatherer economy [1,2]. The early use of pottery, the trends towards sedentism reflected in the settlement pattern, the active management of plant resources, and the possible presence of social stratification are just some of these aspects that have been extensively discussed in the archaeological and anthropological literature [3–6]. While some of these traits do indeed contribute in defining an overall picture of this prehistoric culture, it is undeniable that the Jomon period was far from homogenous, with substantial variation of these traits in space and time. Settlement pattern, the degree of residential mobility, and subsistence economy saw continuous shifts that have been often linked to concurrent changes in climate [7–8] (but see also [9–10]).

Given these premises, it is not surprising that Japanese archaeologists have long been trying to reconstruct Jomon population dynamics from the archaeological record in order to identify relationships with changes in the subsistence-settlement system. Early studies in the 1960s [11] have identified major regional differences in the number of archaeological sites attributed to the Jomon culture, with the northeastern portion of the Japanese archipelago showing a considerably higher density than the southwest. This led scholars, such as Yamanouchi [12], to suggest that this pattern was reflecting the underlying heterogeneity in the distribution of key resources such as acorn, chestnut, and salmon (see also [13]). These early studies did not, however, seek to reconstruct temporal changes in the Jomon population size in a systematic manner, an endeavour that was subsequently pursued by Koyama [14–15]. His analysis confirmed and added more detail to these earliest impressions of spatial variability, providing at the same time a diachronic perspective on Jomon population change. Albeit based exclusively on sites counts and framed by a relatively coarse temporal (archaeological periods of ca 1,000 years) and spatial (regions between 30,000 and 80,000 km^2^) resolutions, the scale of Koyama’s study is still unmatched, and its estimate of absolute population sizes remains the sole attempt proposed so far.

Subsequent studies have offered more detailed, yet fragmented images of Jomon demography. Most have focused on smaller regions, sacrificing the broad comparative dimension offered by Koyama’s work. While site-counts were, and still are, used as proxies, several authors also offered time-series of pit-dwellings [7,16–17], arguing that these provide a more dependable alternative as well as the possibility to rely on a more refined pottery-based chronology, occasionally offering even sub-century temporal resolution [18]. In many cases, these studies have identified fluctuations occurring at scales that were not visible in Koyama’s analysis, prompting many to look at potential correlation with climate change [7–8,19].

While these studies offer insights on Jomon population dynamics, their inferential power is to some degree limited by: 1) a substantial lack of statistical evaluation for distinguishing genuine fluctuations from sampling error; and 2) an almost exclusive reliance on a relative chronological framework, rather than absolute calendar dates. Given the large-scale investment in rescue archaeology and a long tradition of pottery typological studies, it is undeniable that the latter offers an economically viable option with robust sample sizes (*n*>1,000) and the possibility to rely on a fairly detailed sequence (i.e. more than 50 phases for ca 10,000 years). Yet, sample units (i.e. sites and/or residential features) are indirectly dated based on the recovery of diagnostic artefacts, and hence the assignment of the former to specific chronological blocks (periods or phases) is constrained by the quality and the quantity of the latter (see [20,21] for discussion). In many instances, raw data have mixed quality and resolutions and time-series generated from these do not adequately quantify the degree of temporal uncertainty (but see [21]). Furthermore, studies attempting to build an absolute chronological referencing for Jomon pottery phases are still rare and the few exceptions [18,22–24] do not formally address the question of the shape of the underlying distribution of ^14^C dates associated with specific phases (cf. [25]), nor attempt to quantify the uncertainty of the retrieved parameters (as in [26–27]). Consequently, cross-regional comparisons are strongly affected by the proposed matching of different pottery-phase sequences, and the comparison with environmental and climatic data are hindered by chains of indirect dating.

**Fig 1:**
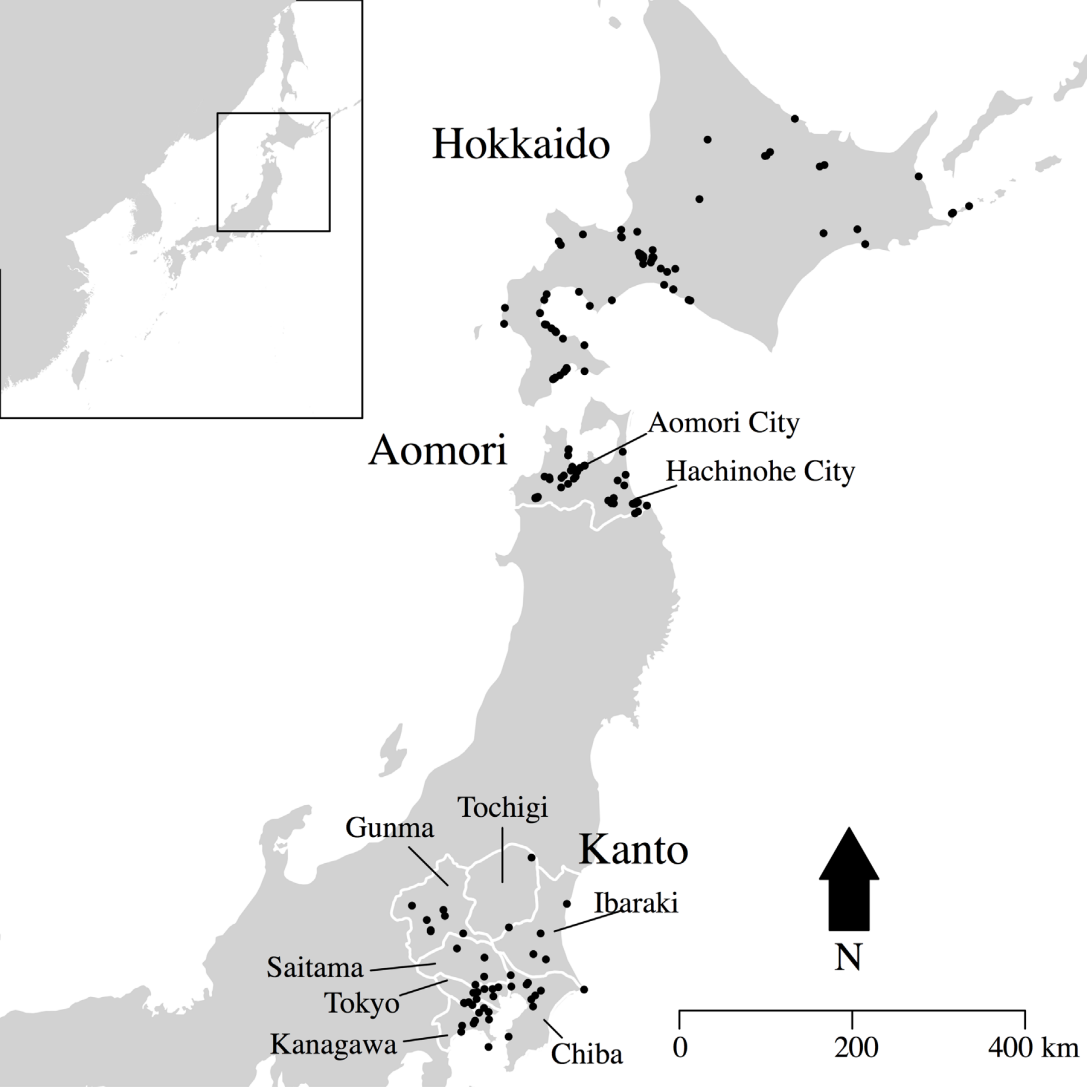
Location of sample sites.

This paper seeks to overcome some of these limitations by using summed probability distribution (SPD) of ^14^C dates to infer Jomon population dynamics. We argue that while counts of pit-dwellings might offer a more direct proxy, the limits imposed by its reliance on a relative chronological framework urge the exploration of alternative proxies. We analyse the SPDs of three regions from Eastern Japan (Hokkaido, Aomori Prefecture, and Kanto; see Fig 1) that were densely populated during the Jomon period: to determine whether key fluctuations are genuine or the result of sampling error and to identify possible divergences in the population trajectory of the three areas within an absolute chronological framework. In order to achieve these objectives, we adopt a hypothesis-testing approach for assessing the SPDs of ^14^C dates, introducing a new permutation-based technique apt to statistically compare different datasets.

### Jomon Population dynamics as inferred by site and residential unit counts

As mentioned, the temporal framework of Jomon archaeology is rarely based on absolute chronology and in most cases it relies on pottery-phases or broader periodisations. Here we briefly review existing attempts of population reconstruction in the three study areas examined in this paper, with reference to such relative temporal frameworks. Although a conclusive agreement is not established, Kobayashi [23] has examined ^14^C dates associated with many of these pottery phases as well as all major Jomon periods, and proposed an absolute chronological sequence. Unless otherwise stated, in the paper we use this chronological reference. In particular, we focus on the interval between 7,000 and 3,000 cal BP, which approximately corresponds to the Early (6,950 ~ 5,470 cal BP), Middle (5,470 ~ 4,420 cal. BP) and the Late (4,420 ~ 3,220 cal BP) Jomon periods. It is worth pointing out that although this scheme is used as a broad chronological reference for eastern Japan (see for example [17]), the ^14^C dates underpinning Kobayashi’s study were primarily retrieved from Kanto, hence the synchronicity of the cultural phases of the three regions is not warranted [18, 28–29].

Early studies [7] based on time-series of dwelling counts from western Kanto (Saitama, Tokyo, and Kanagawa Prefectures) suggest a pattern of three episodes of population rise and fall with different degrees of magnitude. More specifically: 1) a growing trend towards the second half of the Early Jomon period; 2) an abrupt decline in the transition to Middle Jomon; 3) a population maximum towards the second half of the Middle Jomon; 4) followed again by a sharp decline in the transition to the Late Jomon; and 5) a second smaller rise and fall during the middle part of the Late Jomon. A recent statistical reassessment of the same dataset [21] that integrates Kobayashi’s absolute scheme [23] indicates a statistically significant increase in the number of residential units at 5,600–5,400 and 5,100–5,000 cal BP, a significant drop between 4,600 and 4,400, and the timing of the maximum peak at ca. 4,750 cal BP (see also S1A and S1B Fig). Although confined to smaller areas, other studies have confirmed these general trends [9,18,30].

Evidence from Aomori Prefecture and Hokkaido are limited compared to Kanto, primarily because the chronological framework is mostly confined to the scale of period rather than pottery phases. In Hokkaido (S1C Fig), the number of sites increases from the Early (n = 966; 6,950~5,470 cal BP) to Middle Jomon (n = 2,766; 5,470~4,420 cal. BP), and subsequently declines and stabilises during the Late (n = 1,598; 4,420~3,220 cal BP) and Final Jomon periods (n = 1,462; 3,220~2,300 cal BP) [31,32]. In Aomori Prefecture (S1D Fig), the general trend seems to be slightly shifted with the peak of site counts recorded for the Late Jomon period, and the Early, Middle and Final Jomon showing similar and lower figures [17]. The number of residential units shows, however, a different picture (S1E Fig), with a peak during the Middle Jomon followed by a decline during the Late Jomon period, suggesting a general reduction in settlement sizes. A chronologically more refined study in the Hachinohe city area (southeastern part of Aomori Prefecture; Fig 1 and S1 Fig) seem to locate the peak in the dwelling counts between the end of the Middle Jomon and the beginning of the Late Jomon period, followed by a gradual decline throughout the Late and the Final Jomon periods [33].

Broadly speaking, existing time-series based on both site and residential unit counts do seem to indicate similarities in the shape of the population trajectories of these three regions, with a general rise and fall pattern observed at the coarsest temporal scale. However, closer examinations of the data seem to suggest possible divergences. Some fluctuations are observed in certain locations but not in others (e.g. the Early to Middle Jomon decline in the residential unit counts observed in Kanto but not in southeastern Aomori Prefecture), while the timing of specific events such as the major Middle Jomon decline seems to differ (i.e. occurring at the end of Middle Jomon in Kanto and at the middle of the Late Jomon period in southeastern Aomori Prefecture). These observations are, however, constrained by the fact that the chronological subdivision is often at its coarsest scale (e.g. as in Hokkaido), and even when finer resolutions are available the synchronicity of the phases are not warranted. Here we evaluate whether evidence from the SPD of ^14^C dates confirms the presence of a general rise and fall, whether we can identify statistically significant divergences between the three regions and, if so, when these occurred.

## Methods

The last few years saw an exponential increase of archaeological studies that go beyond the simple notion of ^14^C as a dating tool, and seek instead to measure less tangible events, such as the rise and fall of cultural phases [25], the timing of colonisation events [34], or the spread of farming [35]. The use of SPD of ^14^C dates as proxy of ancient demography, for instance, has seen a wide range of applications in Europe and North America, offering new insights and details on the population dynamics of prehistoric societies. In particular, the possibility to rely on absolute calendar dates, rather than the conventional relative chronology offered by the majority of the archaeological record, is giving the unique opportunity to assess the relationship between population dynamics and climatic change [36–39].

In over two decades of application, the sum of ^14^C dates as proxy of prehistoric demography has experienced many criticisms, stimulated heated debates, and promoted revisions and solutions that testifies to the strong interest in the technique and its output. Earliest works based on the sum of uncalibrated ^14^C dates [40–41] pioneered the core assumption of the technique (“more people = more sites = more dates”), and was followed by studies using larger samples and calibrated dates [42], as well as more sophisticated procedures such as the correction of “wealth-bias” by means of pooled mean dates per site-phase [43], or the de-trending of the effects of taphonomic loss [37]. Mere qualitative assessment of the SPDs has been strongly criticised, as in many cases it promoted unwarranted inferential leaps without a sufficiently robust statistical support. Specifically, it has been argued that idiosyncrasies of the calibration curve, small sample sizes, and taphonomic loss can all determine spurious patterns that are *visually* impossible to distinguish from genuine patterns of variation in the density of ^14^C dates [44,45]. Some of these problems have been recently solved by means of a hypothesis testing approach introduced in Shennan et al [36], and further developed in Timpson et al [46]. The core contribution of these works is a shift from a qualitative description of the SPDs to a statistical testing framework, where the empirically observed data are compared against a statistical confidence envelope of a null model. Thus, observed SPDs are first fitted to a generalised linear model, and random calendar years dates are generated via Monte-Carlo simulation and subsequently “back-calibrated” into ^14^C dates to generate null SPDs (see [46] for a detailed description of the technique; further application of the same method can be found in [39] and [47]). This approach allows the detection of statistically significant local deviations (i.e. portions of the SPD showing smaller or greater SPD than the null model) as well as a global significance test. The flexibility offered by Monte-Carlo simulation can further be extended allowing for instance to determine whether gaps in dates are genuine (e.g. [48] for a model based on Poisson distribution), or to quantify the expected consequences of the changing steepness in calibration curves [49].

Here we introduce a non-parametric extension of the hypothesis-testing approach that enables the statistical comparison of two or more sets of ^14^C dates. The null-hypothesis in this case is the equality of the SPDs: the sample dates are generated from identically shaped population curves. Several recent works have relied on the comparison of SPDs. For example, Collard and colleagues [50] have compared different site types, whilst Stevens and Fuller [51] suggested a failing of Neolithic farming in Britain based on the observed divergence in the SPD obtained from hazelnut/wild plants and cereal/crops. We argue that these types of studies can benefit from a statistical test that can highlight statistically significant differences, as well as provide a global *p*-value.

The core principle of our approach can be summarised in the following steps:

1. ^14^C samples of each site of each set are calibrated and aggregated (i.e. a pooled mean is computed) in *bins* based on prior archaeological knowledge (e.g. same context unit) or chronological proximity (e.g. by site-phase, as in [43], or [46]).
2. Pooled mean probabilities obtained from bins are summed to generate an empirical SPD for each set.
3. The assignment of each bin to a specific set is randomly permuted (so that the total number of bins associated to each set is identical to the observed), and an SPD is generated from each set.
4. Step 3 is repeated *n* times, and a local Z-score computed to remove the effects of short term wiggles and the underlying trend of the null model for both observed and simulated data.
5. A 95% upper and lower confidence interval is then computed from the simulated SPD. Observed SPDs above (or below) the envelope is identified as statistically significant local deviations, indicating divergences between the focal set and the aggregate of all sets.
6. Following the same procedure detailed in [46], we generate a null distribution from the total area outside the confidence envelope for each simulated SPD. We then apply the same procedure for each observed set, and compare its value to this distribution. The proportion of the latter that is larger or equal than the observed provides an estimate of the *p*-value for each set. Notice that the comparison is based on the overall shape of the SPDs. In other words, such a global *p*-value might be high even in case significant local deviations are detected, especially when two sets exhibit similar shape for large portions of theirs SPDs.

The approach is robust to inter-regional differences in the research intensity (hence sample size), as the comparison is based on the “shape” of the SPDs (i.e. the relative change in summed probabilities within each region) and not on differences in their absolute magnitudes. As for other frequency-based proxies (e.g. site and house counts), without a quantifiable knowledge of research intensity it is virtually impossible to distinguish whether observed difference in density is due to the actual underlying populations or just a consequence of differences in the sampling fraction. By maintaining the observed number of bins for each region, and by comparing population trajectories rather than absolute differences in density, the proposed method bypasses this problem. Thus, it is worth noting that significant negative (or positive) deviations of the SPD in one region does not necessarily imply a lower (or higher) absolute population density, but that the drop in the proxy within the dynamics of that region was significantly stronger compared to rest of the data.

We first assessed whether the SPD of ^14^C dates for each area showed statistically relevant fluctuations when compared against the uniform and the exponential null models, following the procedure described in [46], using 10,000 Monte-Carlo simulations, and calibrating (via direct numerical integration) with the IntCal13 curve [52] and scripts based on the *Bchron* package [53] in R statistical computing language [54]. The exponential distribution was used as a null model portraying both the temporally increasing taphonomic loss and the long-term population increase observed in prehistoric populations [36,55]. We also examined a uniform distribution as an alternative null that does not assume an exponential increase in the underlying population, but instead look for significant deviations from a simpler “flat” model. The choice of this second null model was partly dictated by a general impression of a rise-and-fall pattern that is distinct from other studies where a steady growing trend is evident (e.g. [36]). We compared the shape of the SPDs of three regions with the permutation test described above, using the same calibration procedure and same number of iterations (i.e. 10,000).

## Materials

We collected over 2,500 ^14^C dates from nine different prefectures: Tokyo, Kanagawa, Saitama, Chiba, Ibaragi, Tochigi, and Gunma (aggregated as Kanto region), Aomori, and Hokkaido (Fig 1). From this initial dataset we kept only samples where the dating was based on Accelerator Mass Spectrometry (AMS). We also removed ^14^C dates based on marine samples (i.e. shell and fishbone), as well as charred remains with high δ^13^C that might be potentially affected by a reservoir effect (i.e. >-24‰, given a range –24.0 ± 0.7 for terrestrial mammals and –25.9 ± 2.0 for C3 plants presented in [56]; a more conservative approach with a threshold set at -26‰ did not show major qualitative differences in the results, see S1 File). We excluded sites dated to Incipient and Initial Jomon periods (when the degree of sedentism was significantly lower), and those associated with the Final Jomon (when the overall sample size was too small in the Kanto region). We thus limited our analysis between the Early and the Late Jomon period, excluding all ^14^C dates outside the interval 7,500 ~ 2,500 ^14^C years BP. The final sample size (n = 1,433; see Fig 1 for site distribution) used for the analysis was 406 ^14^C dates from 47 sites in the Kanto region, 432 ^14^C dates from 58 sites in Aomori Prefecture, and 595 ^14^C dates from 82 sites in Hokkaido. In order to reduce the effect of “ascertainment” or “wealth” bias we binned the ^14^C dates based on the clustering of the mean ^14^C years BP, using a threshold of 200 years (cf. [46]). The resulting number of bins was 87 for Kanto, 128 for Aomori Prefecture, and 186 for Hokkaido. Data, source codes and scripts used in this work can be found in S2 File and in the zenodo online repository (doi: 10.5281/zenodo.47339).

## Results

**Fig 2:**
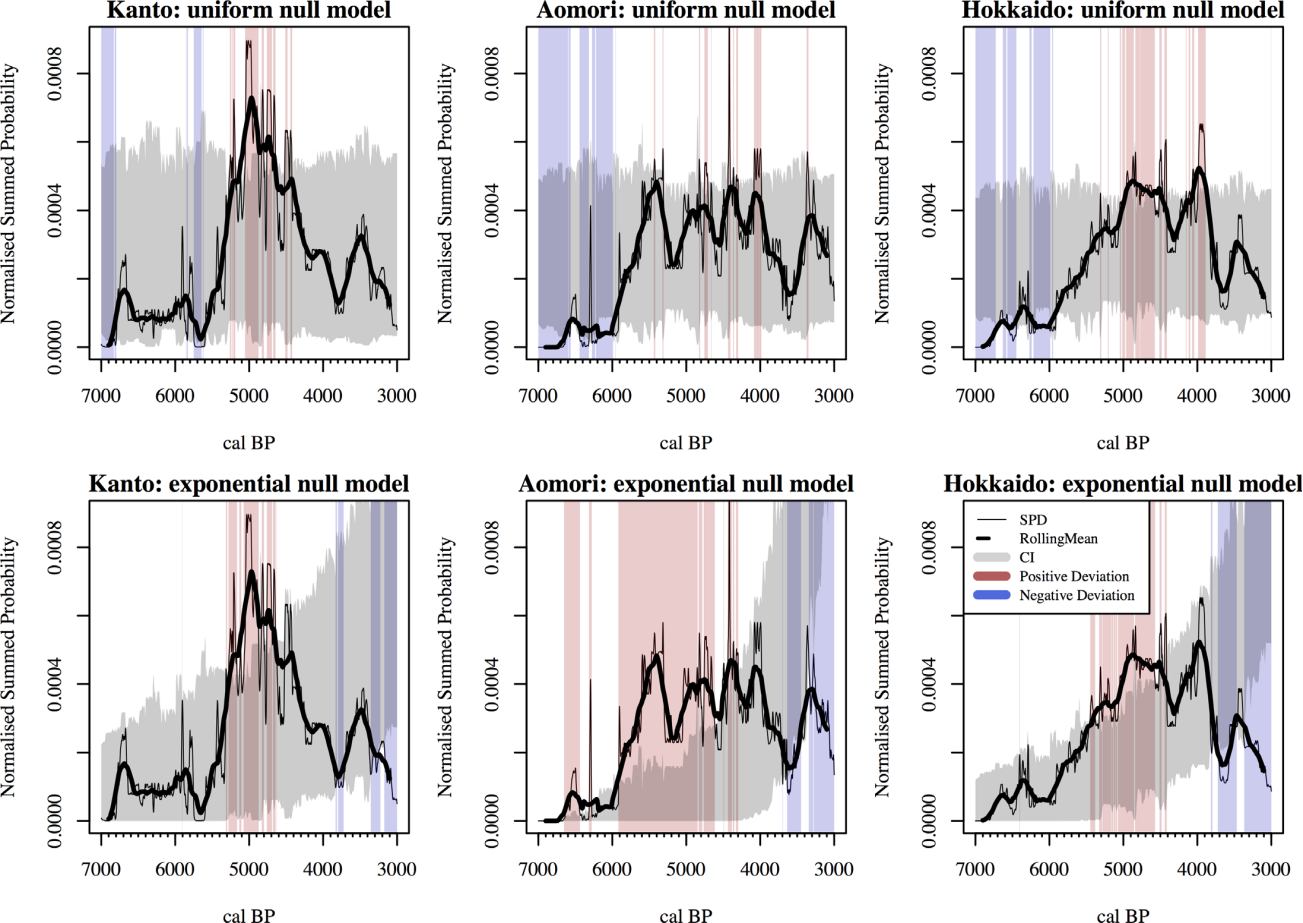
Summed Probability Distribution (SPD) of the three regions (solid line). The thick lines show the 200-years rolling mean, whilst the grey band represents the 95% confidence interval for the null model (upper row: uniform distribution; lower row: exponential distribution). Red and blue vertical bands represent intervals with significant positive and negative deviations.

The statistical comparison of the observed SPD of each study area against their respective fitted uniform and exponential null models (Fig 2) yielded a statistically significant global *p*-value (Table 1), indicating that some of the observed fluctuations are genuine, and not the result of sampling error, taphonomic loss, or a gradual exponential population increase. Local deviations from the null models highlighted in all regions a general rise and fall pattern, with a steady growth starting around the beginning of the 7^th^ millennium BP, and general decline peaking towards the mid 4^th^ millennium BP (Fig 2). In the Kanto region, the population drop during the transition from the Early to Middle Jomon period is confirmed in the statistical comparison with the uniform model, with significant negative deviations around 5,700 cal BP. When compared against the exponential model, this decline is no longer significant, but the overall gradual decline starting after 5,000 cal BP leads the density of ^14^C dates below the expectations of the null model at 3,900 cal BP first, and subsequently, after a temporary recovery, at 3,300 cal BP.

**Table 1:** Significance levels of the global statistics for the null model and the pair-wise permutation tests (values in bold are significant at 0.01, values in italic at 0.1.

	Null Model Test		Pairwise Permutation Test		
	*Uniform*	*Exponential*	*Vs*. *Kanto*	*Vs*. *Aomori*	*Vs*. *Hokkaido*
**Kanto**	**0.0003**	**<0.0001**		0.1596	0.4882
**Kanto (7k-4.42k)**	-	-	-	0.1227	0.667
**Aomori**	**0.0025**	**<0.0001**	*0*.*0555*	-	1.0000
**Aomori (7k-4.42k)**	-	-	**0.0039**	-	1.0000
**Hokkaido**	**<0.0001**	**<0.0001**	0.2752	0.932	-
**Hokkaido (7k-4.42k)**	-	-	0.1124	1.0000	-

In Aomori Prefecture, the overall trajectory delineated by the SPD resembles a logistic curve, with the density of ^14^C dates reaching an upper threshold at 5,500 cal BP. The subsequent interval between 5,500 and 4,000 seems to be relatively stable, with several minor fluctuations deviating from the null uniform model. When compared against the exponential model, the initial moments of this high-density stage is highlighted as significant positive deviation, as well as the temporary (and relatively sharp) drop observed around 3,800–3,700 cal BP.

Finally, in Hokkaido the SPD highlights a steady, uninterrupted growth from c 6,000 cal BP to 5,000 cal BP, followed by a temporary small drop at ca. 4,400 cal BP (albeit not a significant deviation from either of the two models), a short recovery peaking at 4,000 cal BP (significant against both null models), and a more consistent decline with the lowest density of ^14^C dates recorded around 3,700 cal BP (significant deviation against the exponential but not the uniform model).

Some general similarities can be identified from these results. When compared against a uniform model, all three regions show most episodes of negative divergence before c 5,000 cal BP, and most of the positive divergences afterwards. When compared against exponential models the similarities between the three regions are even more striking. In all cases we see positive deviations between 6000 and 5,000 cal BP and a negative deviation after 3,700 cal BP. The results are thus consistent with a general rise-and-fall pattern, where the density of ^14^C dates after the peak is higher than what is observed before.

**Fig 3:**
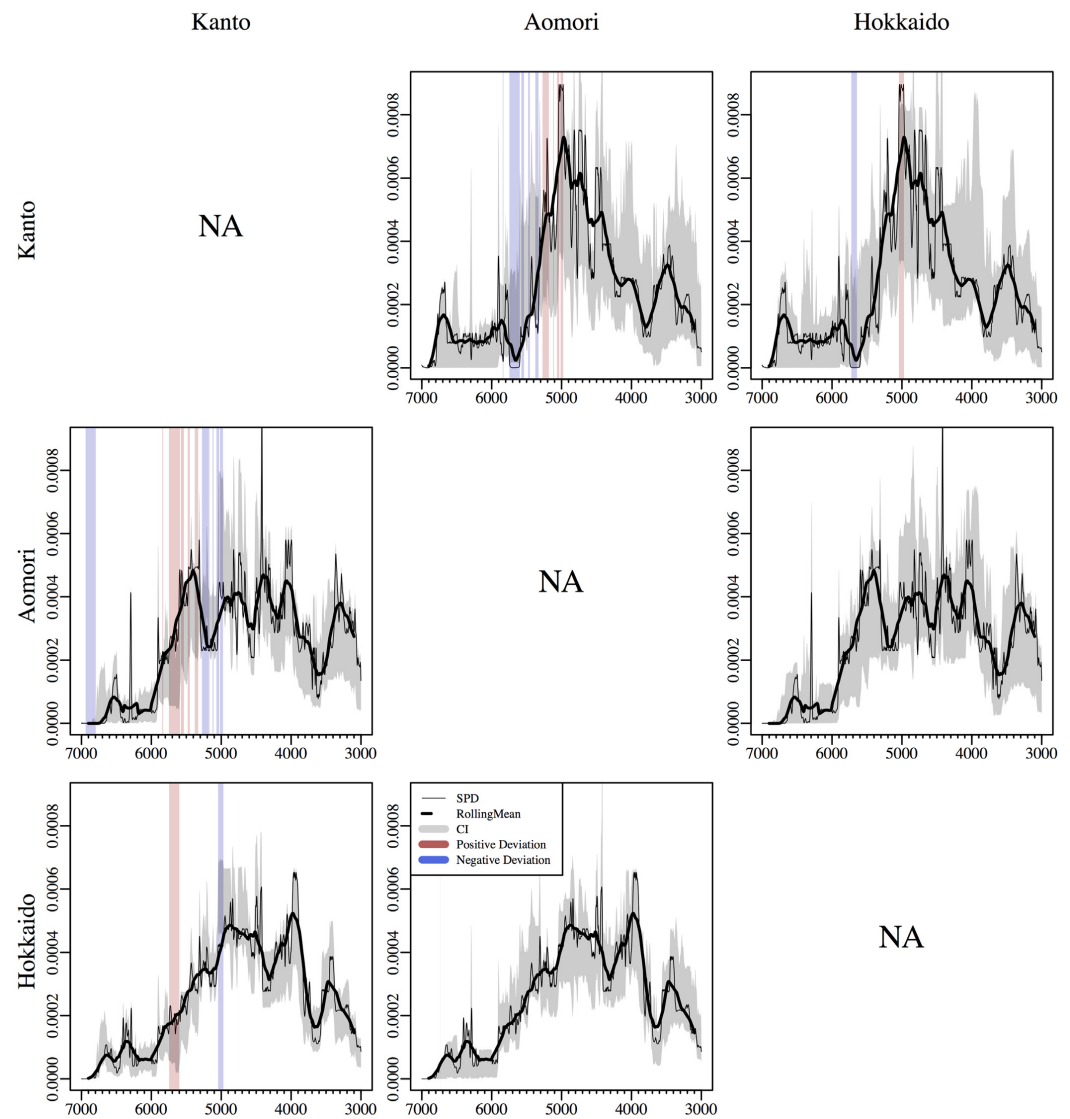
Comparison of the three SPDs. Each row represents an observed SPD of a region compared against another in the column. Red and blue vertical bands represent intervals with significant positive and negative deviations from a null model of the aggregated set of each of pair. The x-axes are in cal BP.

Despite these broad similarities, our analyses indicate the presence of some local divergence in the SPDs. Fig 3 and Table 1 shows the output of the non-parametric pair-wise permutation tests. The global *p*-values (Table 1) are mostly non-significant, albeit the comparison between Aomori and Kanto returned a *p*-value of 0.0555. The local analysis (Fig 3) highlights portions of the SPDs where we observe significant divergences. While none of the divergences between the SPDs of Aomori and Hokkaido can be statistically supported (the global *p*-values are both >0.9), both regions exhibit some significant differences with Kanto. Aomori shows higher density in ^14^C dates around 5,700 cal BP (corresponding to the decline observed in Kanto between the end of Early Jomon and the beginning of Middle Jomon), and a drop in density around 5,200 cal BP (when the SPD in Kanto is showing a steady growth). Although the global *p*-value of Hokkaido against Kanto is non-significant (*p* = 0.2752), we observe local positive deviations again at 5,700 cal BP, and a negative deviation at 5,000 cal BP, corresponding to maximum peak of ^14^C dates in Kanto. Given that all local deviations were in the first 2,000 years, and that the SPDs exhibit substantial similarity during the last 1,500 years, we executed the global significance test reducing the temporal scope to the Early and Middle Jomon periods (7,000~4,420 cal BP, [23]; Table 1). This led to a highly significant divergence between Aomori and Kanto (*p* = 0.0039), but not between Hokkaido and Kanto (*p* = 0.1124). In all cases when the focal set was Kanto the p-values increased (*p* = 0.1227 against Aomori and 0.667 against Hokkaido), most likely due to the smaller sample size of this study area.

## Discussion

Our study provides the first application of SPD analysis for Jomon data, allowing a formal re-assessment of its population dynamics as suggested by the density of ^14^C dates. The case studies offered an exceptionally high density of samples, with a total sample size (n = 1,433) comparable to much larger, continental scale study areas (cf. [39,44]). In qualitative terms, the results confirm some of the general trends suggested by previous works [7,17,21,57] but, more importantly, offers an absolute chronological framework for establishing the timing of key events. Regrettably, the high degree of chronometric uncertainty for sites and pithouses makes the statistical comparison with SPDs unfeasible, as genuine similarities or dissimilarities cannot be distinguished by spurious patterns determined by calibration wiggles and sampling error. We however review our results in relation to existing studies as this can still offer some insights to Jomon population dynamics, but we stress the fact that these comparisons are limited to broad scale qualitative accounts.

In the Kanto region, our analysis indicates a significantly lower density of ^14^C dates around 5700 cal BP compared to the other two regions and the uniform null model. This corresponds to the decline in the counts of residential units observed between the end of the Early Jomon and the beginning of the Middle Jomon (corresponding to the interval between the *Moroiso c* and the *Goryogadai 2* phases [7,58–59]; approximately dated to 5,700 ~ 5,380 cal BP [23]). While our SPD show some differences in the timing of the subsequent increase in the population trajectory when compared with previous works (at 5,500 cal BP in our study, later in [9,21]), the matching is quite remarkable, and confirms possible correlation with several environmental changes linked with the Bond 4 event (ca. 5.9 k cal BP [60]; see [19] for a recent discussion). However, in Aomori Prefecture and Hokkaido, this period shows evidence of a steady growth, suggesting that a continental climate-based hypothesis should take into account both local environmental variations and potentially divergent cultural responses in different parts of the Japanese archipelago.

All case studies show evidence of a decline after a period of relatively high density of ^14^C dates associated with the Middle Jomon period (albeit in Aomori Prefecture there is a significant temporary drop in the SPD around 5,300–5,200 cal BP). In the Kanto region, this maximum is characterised as a single, statistically significant peak around 5,000 cal BP, thus few hundred years earlier than what has been suggested in previous works (e.g. [9,18,21]). In Aomori Prefecture and Hokkaido, the patterns resemble more a high-density plateau between 5,500 and 4,000 cal BP, with some statistically significant fluctuations (when compared against the uniform null). In most cases, the pair-wise permutation test does not suggest significant divergences in these fluctuations, although the 5,000 cal BP peak in the SPD observed in Kanto showed a significantly higher density compared to the other two regions.

The comparison of the three SPD does, however, not indicate any statistically significant divergence after 5,000 cal BP. The timing of the decline of the general rise-and fall pattern seems to have occurred slightly earlier in the Kanto region (right after 5,000 cal BP) compared to the other two study areas (after 4,000 cal BP). The decline observed in the SPD of the Kanto region appears to be matching the lowering in the number of pit-houses and sites dated between the Middle and Late Jomon period (generally linked from the *Kasori* E4 to the *Shomyoji* phases; 4,520 ~ 4,250 cal BP; [23]), but no statistically significant deviations have been observed during this interval (5,000~4,000 cal BP) in any of the regions. It is worth stressing, however, that the fact that we failed to reject the null hypotheses for this stage does not imply that there was no decline. The impression given by the SPD was that the change observed in Kanto was more akin to a gradual return to “normal conditions” (after the peak of 5,000 cal BP) rather than an episode of a sudden “collapse”. This is also in line with some recent studies based on the time-series of residential units and site counts where the absolute density of features remains higher than the conditions observed before the Middle Jomon peak (e.g. [9]).

The SPD of the Kanto Region does however suggest that this slow decline continued, and reached a statistically significant trough (when compared against the exponential model) at ca. 3,900~3,800 cal BP. In Aomori Prefecture and Hokkaido, a similar, and also statistically significant trough, is observed around 3,800~3,500 cal BP. In Kanto, this period would correspond to the *Horinouchi* 2 phase (3,980~3,820 cal BP; [23]), when indeed some evidence of decline is observed from other proxies [9,21]. In Aomori, the observed decline seems to be concurrent to the decline in residential features observed in the Hachinohe city area around the middle of the Late Jomon period, although the lack of absolute dates does not allow us to further assess this claim. This interval is later than the estimated cooling of ~2°C at around 4,100 cal BP in the sea surface temperature of the Mutsu bay area [61], located between Aomori and Hokkaido. Further studies will hopefully clarify the relationship between this cooling event and the population decline identified here.

Determining whether these differences between the SPDs and the other existing proxies (i.e. counts of residential units and sites) are real, or dictated by chronometric uncertainty of the latter, is not trivial. Jomon pottery phases are assumed to be non-overlapping, and hence a decline in the number of features associated to a specific phase will undoubtedly generated a trough in the time-series. While this can be a genuine reflection of the underlying population dynamics, abutting and spatially synchronous archaeological phases is often an unrealistic assumption that might introduce a significant bias, especially when we consider larger study areas. In contrast, the probabilistic nature of SPDs implies that, in order to have a statistical significant trough in the time-series, the duration of the low-density interval must be sufficiently large. Hence a sudden drop and reprise in the population for a period of less than 100~200 years are unlikely to be detected by the SPD, and virtually undistinguishable from the patterns generated by the calibration wiggles. For instance, previous studies have suggested [62] a temporary decline in the number of larger settlements around the end of the Middle Jomon in Aomori Prefecture. The SPD of this region does indeed show a small trough around c. 4300 cal BP, but the signal—possibly weakened by the uncertainty of the calibration process (see [63] for discussions on how different calibration techniques can yield different results at finer temporal resolutions) and idiosyncrasies of the calibration wiggles—was not statistically significant when compared against our two null models. Indeed we agree with several authors [44,46,48] that, when examined visually, SPD should be smoothed and in this case we used a 200-year moving window (thick line in Fig 2 and Fig 3). It is worth noting, however, that the intrinsic limitations dictated by ^14^C dating similarly applies to the chronometric assessments of pottery phases, which is also affected by biases derived by sample size and indirect dating. As it stands, the temporal resolution imposed by this proxy is still the best compromise necessary in order to infer chronological relationship between anthropic and climatic events.

When examined on its own, we can consider a number of alternative causes that might have led to the observed fluctuations in the SPDs. A general time-dependent taphonomic loss [56] would not affect our analyses (the exponential model mimics the process and the permutation-based comparison of the SPDs uses the observed ^14^C dates directly, hence already integrating the effects of time-dependent loss), but a spatially divergent, inhomogeneous thinning process might produce some fluctuations that are not related to the underlying population dynamics. This problem is clearly not limited to SPDs and applies to all count-based time-series, but we are not aware of any study suggesting and quantifying this type of bias.

While our method is not affected by biases introduced by regional differences in research intensity, the SPD could showcase spurious patterns if there is evidence of a temporally heterogeneous dating process. For instance, scholars might focus on dating sites of specific chronological interval rather than others, effectively leading to a higher density of ^14^C dates. We are confident that this research bias is not affecting the samples from Hokkaido and Aomori Prefecture, but the strong interest in reconstructing the Middle Jomon pottery-sequence of the Kanto area might have generated a higher density of ^14^C dates during this interval (see for instance [18]). This might indeed be the reason why we observe a strong positive deviation around 5,000 cal BP in Kanto. However, the fact that we observe similar peaks in other proxies (e.g. counts of residential units) seems to support our argument, which the SPD is genuinely reflecting an increase in population size. Similarly, the preferential dating of specific site-types over others (e.g. exceptionally large settlements) might potentially be affecting some of the pattern we observe in the SPD, albeit the broader similarity to other proxies does not seem to suggest this.

The results of our SPD analysis might be a consequence not only of changes in the underlying population but also the result of variations in the site-to-population ratio. Sites might in fact vary in their function (e.g. settlements vs. field camps), size (i.e. number of residential features), duration of occupation, and archaeological visibility as a result of changes in the subsistence-settlement patterns. Using counts of residential features as basic unit of analysis does not necessarily solve this issue. In fact, one should consider also variations in the size of residential units, and more crucially the patterns of residential mobility. For instance, a change from year-round settlements to seasonal shifts would increase the number of sites (and hence lead to a higher density of ^14^C dates), even if the population size remains unchanged. Similarly, if the seasonal shifts involved fission-fusion of residential units, the average size of the seasonal residential bases could be smaller than the nucleated residential bases of fully sedentary hunter-gatherers.

Our case studies do have evidence of potential changes in the subsistence-settlement pattern during the temporal scope investigated here. In the case of Aomori Prefecture and Kanto, the archaeological evidence from the middle of the Middle Jomon period is characterized by an abundance of extremely large nucleated settlements such as the Sannai Maruyama site in Aomori Prefecture [3,64] and the Miharada site in Gunma Prefecture in Kanto [65,66]. Towards the end of the Middle Jomon period, however, scholars have reported an increase in the number of smaller settlements [17,67]. Similarly, settlement analyses in the Eastern Tokyo Bay area have shown continuous fluctuations in the shape of the site-size distribution between the Early and Late Jomon periods [9]. For an earlier phase, lithic and settlement data seem to indicate that the transitional period between the Early and Middle Jomon in some parts of Kanto was characterised by a temporary shift from a collector to a forager strategy [59]. It is an issue open for debate whether under the assumption of a constant population size these changes in settlement pattern would translate into tangible patterns in the proxies we examined here. If archaeological visibility is higher for all the residential bases, we might expect an increase from a collector to a forager system in terms of site number (forager systems would imply a higher number of residential move for the same interval), and hence also a higher number of ^14^C dates. If the sampling is unbiased (for example, if the sampling is more representative to the full spectrum of other site types such as caches and stations), this might not be the case. Further studies aiming to establish differences in the site-to-population ratio for different settlement patterns is thus a key aspect for the development of SPD analysis.

Finally, from the perspective of Jomon archaeology, our analyses highlight the Middle Jomon rise and fall, which is one of its most intriguing phenomena. If we assume that the SPD of ^14^C dates and the other existing proxies (site and residential units counts) are genuine reflections of the underlying demography, Jomon communities experienced a population growth that is most likely comparable to the Neolithic demographic transition in Europe [68]. While there are currently no comprehensive palaeoanthropological studies similar to those applied in other parts of the world [68–70], a proper evaluation with such an independent proxy is certainly desired. The underlying cause of this potential population increase is still debated, but many [58,62] have noted that there are several lines of evidence suggesting a parallel increase in the reliance on plant resources, possibly coupled with instances of active management and consequent niche construction activities [5,71–72]. It is tempting to hypothesise that the subsequent decline in population size was the result of the subsistence economy failure due to its overspecialisation to fewer resources more susceptible to climatic changes [62]. Although confined to the evidence of a single site, analyses of tool assemblages from Sannai Maruyama in northern Japan do indeed exhibit a subsistence specialisation focused on plant food during a stage of settlement expansion, followed by a decline in the number of residential features and a concurrent decrease of plant exploitation in favour of a more prominent role of hunting [10, 62]. Kawahata and colleagues suggest that this rise and fall of Sannai Maruyama was ‘closely influenced by cyclic climatic changes’ [61]. They also suggest an increase in the marine biogenic production in northern Japan between 5.9 and 4.0 k cal BP [61], an interval that roughly matches the growth and stability interval suggested by the SPD of ^14^C dates (see Fig 2). This evidence is certainly not definitive, but the absolute chronological framework provided by our analysis can set the basis for future studies designed to tackle this correlation (as for instance in [36]), and offers at the same the possibility of a global cross-cultural analysis of prehistoric population dynamics [73].

## Conclusion

The use of SPD for inferring past population dynamics has recently sparked debate, raising questions concerning the reliability of the underlying data and its general validity as a proxy (e.g. [45,74–76]). As it stands, the advantages provided by SPD over alternative methods outweighs its potentials limitations. We however stress that no proxy provides a unique and definitive portrait of prehistoric demography, and that when possible, multiple lines of evidence [70,77,78] should be assessed in comparative fashion. Furthermore, we would like to highlight that SPDs, as well as other count-based proxies, should never be simply assessed in visual terms, but compared against formal statistical models. Future studies should also go beyond the hypothesis-rejecting framework, and aim to statistically compare multiple working hypotheses. Recent advances in likelihood-free inferential methods such as Approximate Bayesian Computation [79–80] is offering potential new lines of research that has already been applied for the study of prehistoric demography [47].

The SPD of the ^14^C dates from Hokkaido, Aomori, and the Kanto region do exhibit patterns that closely match other lines of evidence [14,57], offering at the same time an absolute chronology of key demographic events pertaining to the general rise-and-fall pattern observed in northeastern Japan. In particular, the number of ^14^C dates exhibits a rapid exponential increase between 6,000 and 5,000 cal BP, followed by a high-density interval between 5,000 and 4,000 cal BP, a decline with a trough at c. 3,700 cal BP, and a renewed growth in the subsequent centuries, peaking at c. 3,300 cal BP. In broad terms, this pattern is consistent with the idea of a higher population density between the Bond 4 (5,900 cal BP) and 3 (4,200 cal BP) events [60], although further studies based on local climatic proxy will be necessary to establish this association (cf. [8,61]).

Albeit all three areas share broad similarity in their population history, our analysis also indicates that the SPD of the Kanto region show two significant divergences when compared to the other two areas: a temporary decline at c. 5,700 cal BP, and a peak at c. 5,000 cal BP immediately followed by a continuous decline till 3,700 cal BP. This is in contrast to a more stable “plateau” pattern during the 5^th^ millennium cal BP, and a decline starting only at 4,000 cal BP observed in the Hokkaido and Aomori regions. In general terms, these patterns support previous studies based on the time-series of pit-dwelling counts, but at the same time overcome many of the limitations imposed by a relative chronological framework. The SPD analysis’ major results are the ability to define the absolute timing of these events, as well as substantiating that these regional divergences are not due to simple mismatches in the relative pottery-based chronological sequences nor to sampling errors. Establishing how much of the observed differences in the SPDs are due to underlying divergences in the population history and how much to variation in subsistence-settlement strategies remains, however, an intriguing open question. A combination of multi-proxy demographic analyses with independent lines of evidence, a comparative assessment of the subsistence data, and formal evaluation of the temporal relationship with climatic changes are the next key directions to be undertaken in this regard.

## Supporting Information

S1 Fig Time-series of pithouse and site counts. Time-series of pithouse and site counts: a) pithouse counts in Saitama, Kanagawa, and Tokyo prefectures (95% confidence intervals obtained from 1,000 Monte-Carlo iterations; details in [21]); b) rate of change estimates of pithouse counts in Saitama, Kanagawa, and Tokyo prefectures (95% confidence intervals obtained from 1,000 Monte-Carlo iterations; details in [21]); c) site counts in Hokkaido (data from [31]); d) site counts in Aomori (data from [17]); e) pithouse counts in Aomori (data from [17]); f) pithouse counts in the Hachinohe City area (data from [17,33]).
(PDF)

S1 File Results of the SPD analysis using^14^C dates with δ^13^C < -26‰. (PDF)

S2 File R Source Code, Scripts, and Data. (ZIP)
