# Supplementary figures and images for: Summed Probability Distribution of 14C Dates Suggests Regional Divergences in the Population Dynamics of the Jomon Period in Eastern Japan
Source: PLoS One. 2016 Apr 29;11(4):e0154809. doi: 10.1371/journal.pone.0154809 (PMC4851332; doi:10.1371/journal.pone.0154809)

**a**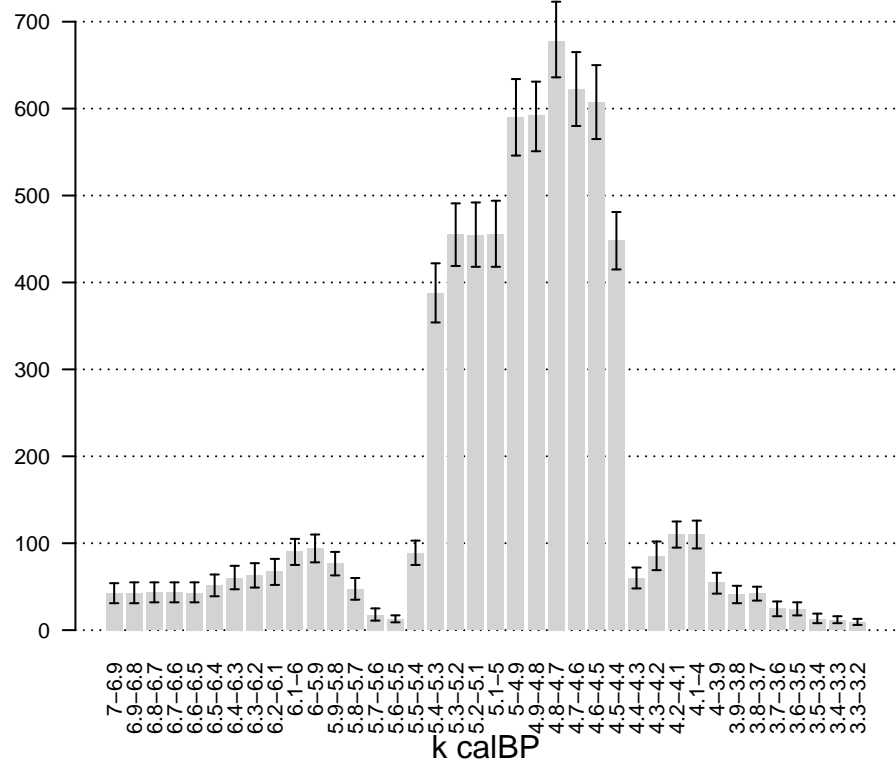**c**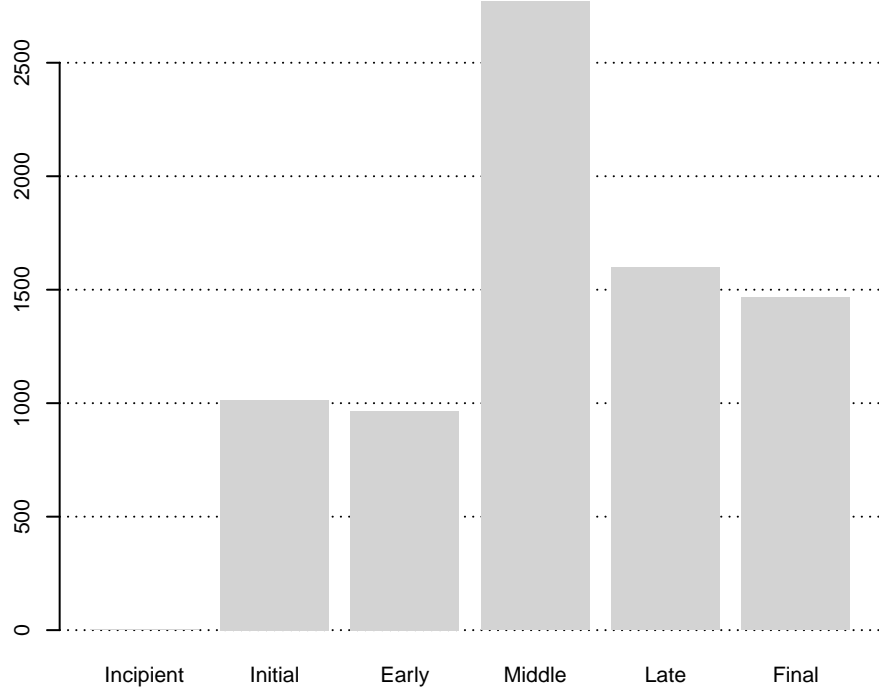**e**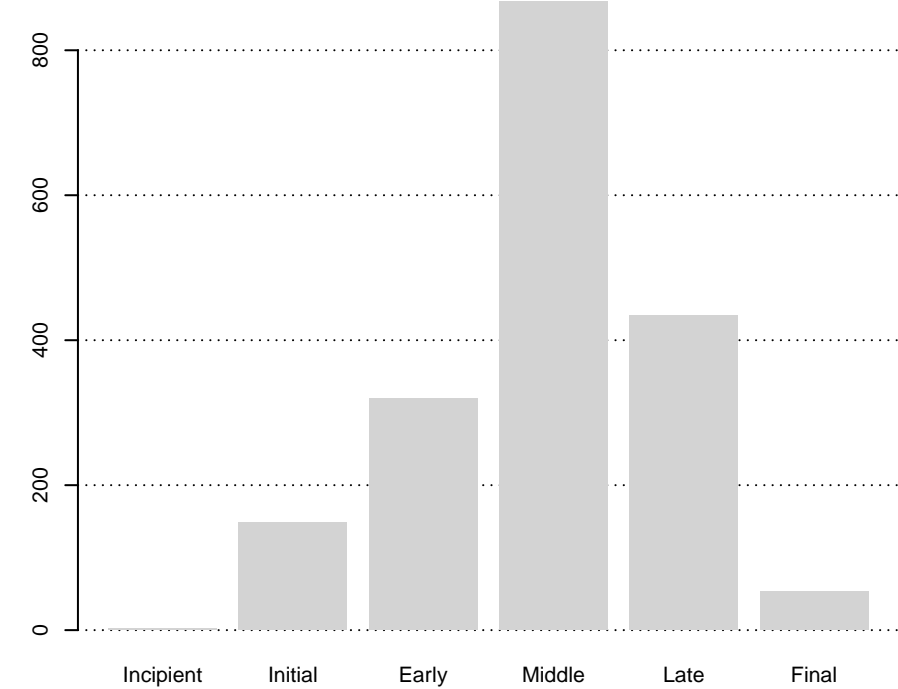**b**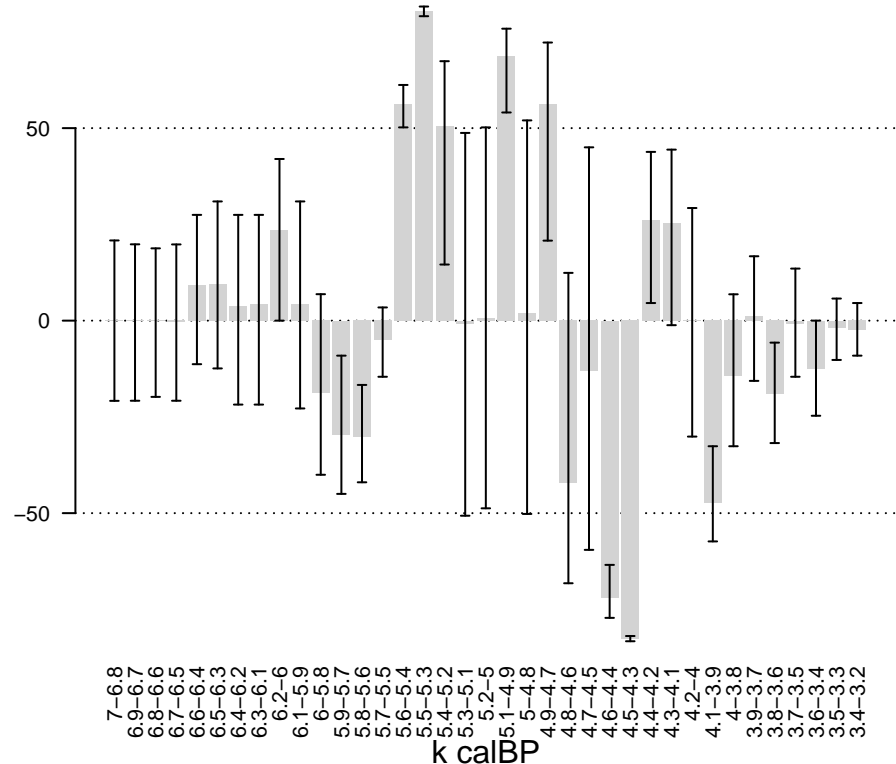**d**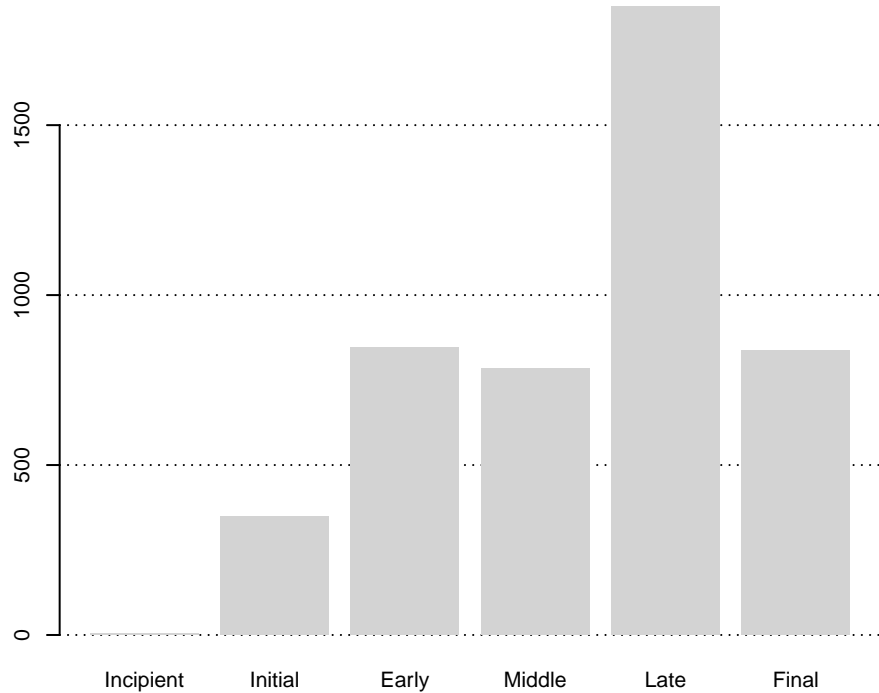**f**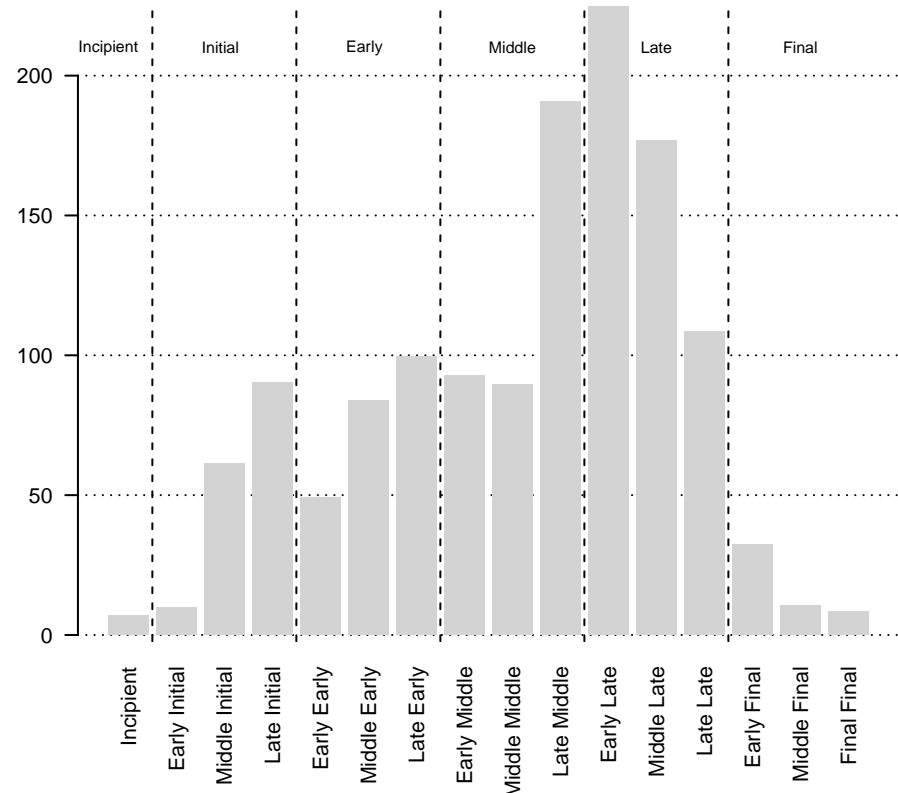

Supplement: S1 Fig — Time-series of pithouse and site counts: a) pithouse counts in Saitama, Kanagawa, and Tokyo prefectures (95% confidence intervals obtained from 1,000 Monte-Carlo iterations; details in [21]); b) rate of change estimates of pithouse counts in Saitama, Kanagawa, and Tokyo prefectures (95% confidence intervals obtained from 1,000 Monte-Carlo iterations; details in [21]); c) site counts in Hokkaido (data from [31]); d) site counts in Aomori (data from [17]); e) pithouse counts in Aomori (data from [17]); f) pithouse counts in the Hachinohe City area (data from [17,33]). (PDF) [file pone.0154809.s001.pdf]
