# Supplementary material for: Summed Probability Distribution of 14C Dates Suggests Regional Divergences in the Population Dynamics of the Jomon Period in Eastern Japan
Source: PLoS One. 2016 Apr 29;11(4):e0154809. doi: 10.1371/journal.pone.0154809 (PMC4851332; doi:10.1371/journal.pone.0154809)

S1 File
for the paper:

"Summed Probability Distribution of ^{14}C Dates suggests Regional Divergences in the Population Dynamics of the Jomon Period in Eastern Japan"

by Crema ER, Habu J, Kobayashi K, Madella M

Sample Sizes **(with using^{14}C dates with δ^{13}C < -26‰):**

N.^{14}C Dates: Kanto (n=276); Aomori (n=259); Hokkaido (n=324);
N. Sites: Kanto (n=41); Aomori (n=48); Hokkaido (n=71);
N. Bins: Kanto (n=75); Aomori (n=90); Hokkaido (n=136);

Global Significance Test:

	**Null Model Test**		**Pairwise Permutation Test**		
	Uniform	*Exponential*	*Vs Kanto*	*Vs Aomori*	*Vs Hokkaido*
Kanto	**0.0083**	**0.0004**		0.1033	0.7772
Kanto (7k-4.42k)	-	-	-	*0.0771*	0.5217
Aomori	**0.0025**	**<0.0001**	**0.0475**	-	1.0000
Aomori (7k-4.42k)	-	-	**0.0106**	-	1.0000
Hokkaido	**<0.0001**	**<0.0001**	0.2708	1.0000	-
Hokkaido (7k-4.42k)	-	-	0.1141	1.0000	-

Local Significance Test (Null Models):

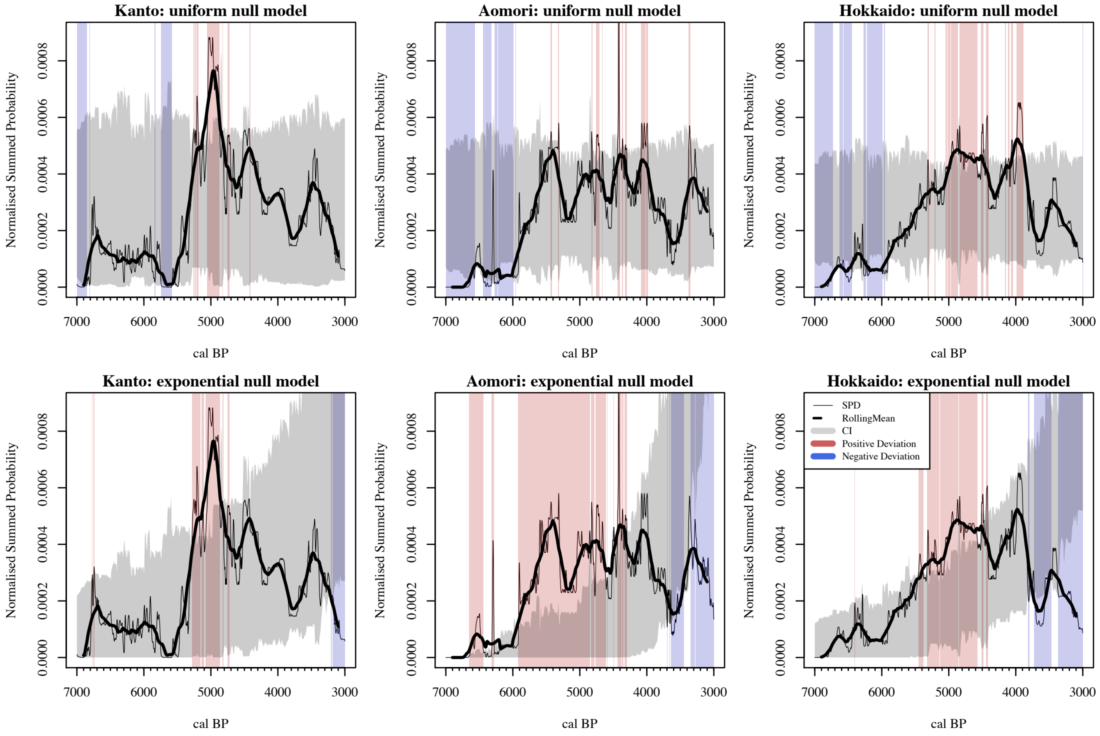

Local Significance Test (Pairwise permutation test)

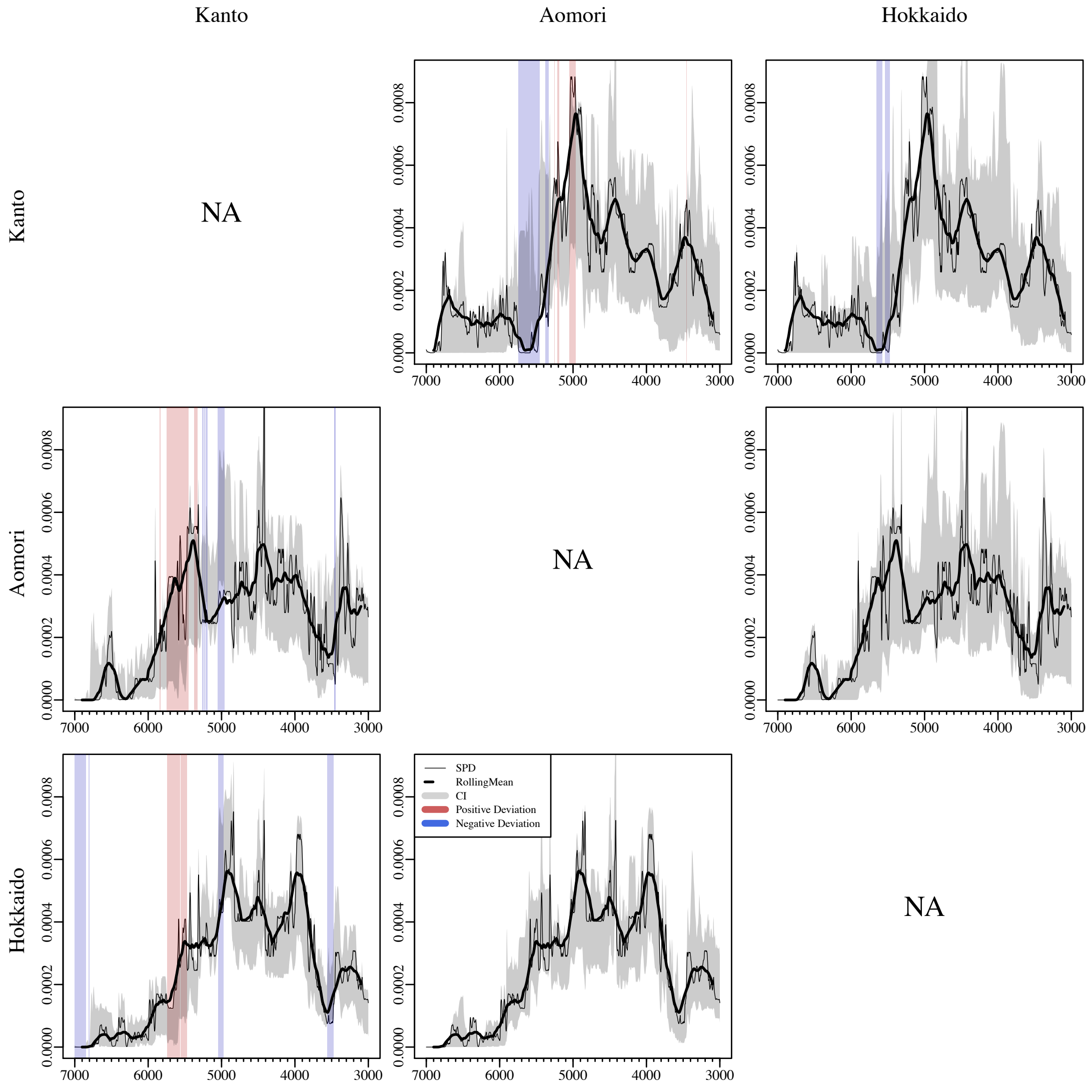

Supplement: S1 File — (PDF) [file pone.0154809.s002.pdf]
